# Sequence effects on mutation rates investigated in whole-genome sequenced UK Biobank participants

**DOI:** 10.1093/g3journal/jkag207

**Published:** 2026-07-29

**Authors:** David Curtis

**Affiliations:** UCL Genetics Institute, U niversity College London, United Kingdom

**Keywords:** mutational signature, trinucleotide context, single base substitution, transcription, strand asymmetry

## Abstract

UK Biobank has released whole-genome sequence data for 500,000 participants, including allele counts for hundreds of millions of variants and these were considered in the context of the pentanucleotide background on which they occurred. Variants with an allele count of 25 were found to closely mirror previously reported de novo mutations (DNMs) in terms of the frequencies of variant types. Therefore these variants, referred to as AC25 variants, were used to investigate factors relevant to mutation frequency. The counts of AC25 variant types in a trinucleotide context could be well approximated by combining 7 mutational signatures previously obtained from studies of cancer cells. Frequencies of variants were strongly influenced by context. C > T variants occurred more often in the CpG context but other features of the trinucleotide context also had marked effects. Although the trinucleotide context was a very strong predictor of the variant frequencies for the full pentanucleotide context, there were examples where the more distant nucleotides did have marked effects. Here, the configuration of bases 2 upstream or downstream could have a more than 2-fold effect on variant frequencies, presumed to reflect mutation rates. For some variants frequencies varied between nontranscribed and transcribed regions and variants with higher frequencies in transcripts of protein coding genes also demonstrated strand asymmetry, being more frequent on the coding than template strand. Genes with high frequencies of these variants showed enrichment for basic cellular processes, meaning that they could have arisen as a result of transcriptional events during embryogenesis prior to entering the germline. Investigating the molecular mechanisms whereby contexts moderate mutation rates could lead to a better understanding of variant pathogenicity and how DNA fidelity is conserved in normal tissue. This research has been conducted using the UK Biobank Resource.

## Introduction

Ultimately, all genome sequences are derived from processes which involve mutation, in which there are imperfections in the molecular processes responsible for copying the genetic code from 1 cell to another. Investigating patterns of mutation may yield insights into these underlying mechanisms. Additionally, it could assist in distinguishing individual variants which are of rare types and hence which might be more likely to be pathogenic (Curtis 2024). In cancers, mutations occur with increasing frequency as the disease progresses and a fruitful area of research has been to study the mutational signatures seen in different types of cancer (Alexandrov et al. 2020). This involves enumerating the counts for each mutation, for example a single base substitution (SBS), specific to the background upon which that mutation occurs, for example the 2 flanking bases. The mutations observed in a sample can be considered to be produced by number of different drivers of mutation involved in DNA damage, repair and/or replication, possibly involving different endogenous and exogenous exposures, and each driver will produce a characteristic mutational signature. Methods have been devised to disaggregate an observed set of mutations into its component signatures (Díaz-Gay et al. 2023).

Although cancers provide a useful source of data involving large numbers of somatic mutations, information can also be derived from other sources. If parents and children are both sequenced then de novo mutations (DNMs) can be observed (Rahbari et al. 2016; Jónsson et al. 2017; McRae et al. 2017). A sample of 2,976 whole-genome sequenced trios was used to identify 200,435 DNMs and these were used to assess the mutation probabilities for different trinucleotides as well as how these were impacted by crossover events (Halldorsson et al. 2019).

Whole–genome sequenced population samples such as 1,000 Genomes can be used to reconstruct genealogies and these can then be used to make inferences about mutational processes and their association with different population features (Gao et al. 2023). Sequencing sperm allows for the investigation of mutation within an individual and can demonstrate the relationship between mutation rate and paternal age (Shoag et al. 2025). An alternative approach is to study polymorphisms observed in a large sample of individuals and in this context it has been reported, for example, that C > T substitutions are seen more often in the context of CpG dinucleotides, an observation which has been explained as being due to the spontaneous deamination of 5-methylcytosine (Zhao and Boerwinkle 2002; Aggarwala and Voight 2016). Effects on substitution rates were also observed for more distant bases, with the heptanucleotide context explaining >81% of variability in substitution probabilities (Aggarwala and Voight 2016). Frequencies of all polymorphisms observed in a population reflect not only ongoing mutational processes but also historic processes, including selection. Instead, attention may be confined to variants occurring only as singletons, on the assumption that these are likely to reflect patterns of recent mutation. In a sample of 3,560 whole-genome sequences for the Bipolar Research in Deep Genome and Epigenome Sequencing (BRIDGES) study, 36 million singleton variants were used to estimate relative mutation rates for different mutation types, which were shown to be influenced by nucleotide context and other genomic features (Carlson et al. 2018).

More recent work has thrown into question the appropriateness of using singleton variants to estimate mutation rates. With a constant population size and a neutral mutation rate *u*, the allele frequency spectrum is such that the expected number of variants to have exactly *i* copies of the alternate allele in a sample is proportional to *u*/*i* under the “infinite sites” assumption that there are no recurrent mutations (Kimura 1969). However for sites with high mutation rates at which recurrent mutations can occur there will be a reduction in low frequency variants and an excess at higher frequencies (Wakeley et al. 2023). This means that the singleton count is low relative to the mutation rate for variant types whose mutation rate is high and so overall the singleton counts across different variant types will not correlate well with their mutation rates. A follow-up study of the BRIDGES samples confirmed that the allele frequency spectrum differed between variant types, with the singleton to doubleton ratio being lower for variant types with higher mutation rates, and showed that this heterogeneity could impact inferences about population genetic history (Liao et al. 2023).

Although mutations can occur through errors in DNA replication or failures of repair mechanisms following damage, transcription associated mutations can be considered separately and these can lead to strand asymmetries (Green et al. 2003; Cui et al. 2012). In order for mutations produced during transcription to be observed in the germline, they must occur in genes which are expressed early in embryonic development or in the lineage of gametogenic cells. Whole-genome sequence data for 500,000 UK Biobank participants has now been released, including hundreds of millions of variants (The UK Biobank Whole-Genome Sequencing Consortium 2025). This large sample was used to obtain estimates of relative mutation rates for different types of variant according to their pentanucleotide context. Mutation rates were compared between transcribed and nontranscribed regions and for transcribed regions additional analyses were carried out to identify mutation types demonstrating strand asymmetry.

## Materials and methods

### Obtaining variant counts

The UK Biobank Research Analysis Platform was used to access the whole-genome sequence data which had been obtained using the methods previously described (The UK Biobank Whole-Genome Sequencing Consortium 2025). UK Biobank had obtained ethics approval from the North West Multi-centre Research Ethics Committee which covers the UK (approval number: 11/NW/0382) and had obtained written informed consent from all participants. The UK Biobank approved an application for use of the data (ID 51119) and ethics approval for the analyses was obtained from the UCL Research Ethics Committee (11527/001). For each chromosome, the *pvar* file was downloaded for the DRAGEN population WGS variants in PLINK format. The *pvar* file contains the coordinate, DNA change and allele count (AC) for each variant but no information about individual participants. Attention was restricted to SBS variants. All analyses were confined to noncentromeric regions because the centromeres include repetitive DNA sequences which are highly homogeneous and hence their sequence content is not representative of the rest of the genome (Altemose et al. 2022). The telomeric regions were also excluded. In the included regions there were a total of 1.06e9 SBS variants, of which 4.05e6 were singletons and 1.48e6 were doubletons.

Each SBS variant was considered in the context of the 2 bases 5′ and 2 bases 3′, so that the fundamental unit of analysis consisted of a pentanucleotide sequence with the variant consisting of a change from reference to alternate for the central base. A variant type is then defined by 6 bases, the pentanucleotide background along with the alternate central base, for example AA[G > C]TG, meaning that there was a total of 4^5 × 3 = 3,072 types. The AC field for each *pvar* file was used to obtain total counts for each variant type binned according to the AC field, consisting of counts of singletons, doubletons, AC = 3, etc. up to AC = 200.

A list of DNMs obtained from 2,786 whole-genome sequenced trios described above was downloaded from https://www.science.org/doi/suppl/10.1126/science.aau1043/suppl_file/aau1043_datas5_revision1.tsv (Halldorsson et al. 2019). Counts of variant types among these DNMs, consisting of the variant in the context of its pentanucleotide background, were enumerated. In the regions excluding the centromeres and telomeres, there were a total of 178,610 SBS DNMs.

For each AC bin, the frequency of each variant type was obtained, consisting of the count divided by the number of times the background pentanucleotide occurred in the reference genome, and the frequencies for the DNMs were obtained in the same way. As previously reported, the ratio of singletons to doubletons was markedly reduced for variant types with higher mutation rates, consisting of those including the [C > T]G motif, and across the 3,072 types the correlation of singleton frequencies with the DNM frequencies was only 0.043. This correlation increased to 0.442 for doubletons and to 0.704 for tripletons and continued increasing with AC. At the same time, the total number of variants in each AC bin gradually reduced. With AC = 25, the correlation between variant frequencies and DNM frequencies across variant types reached 0.990. Thus, the variants which have an AC of 25 almost exactly predict the relative mutation frequencies observed directly in DNMs and these will now be referred to as AC25 variants. Classical population genetics predicts that the expected number of variants to have *i* copies of the alternate allele is proportional to *u*/*i* so that for a particular value of *i* the relative frequencies of variant types are expected to mirror their mutation rates (Kimura 1969). While this breaks down for singletons and other rare variants, presumably because of recurrent mutations, as the AC increases toward 25 it seems that the distortion becomes negligible and the frequencies become accurate indices of relative mutation rates. There were in total 2,654,130 AC25 variants so it was thought that this sample, much larger than the 178,610 DNMs, could be used to obtain more accurate estimates of effects of sequence on mutation probability than had been possible with the DNMs.

### Mutational signature analysis

Previous investigations of mutational signatures in cancer have used counts of variants occurring on a trinucleotide background sequence and use the 96 variant types having C or T as the central base, using the sequence on the minus strand when the central base on the plus strand is G or A (Alexandrov et al. 2020). These counts depend on both the mutation rate and the number of times the background sequence occurs within the genome. Hence, the AC25 counts for the variant types based on pentanucleotide backgrounds were used and were pooled according to this scheme using the central trinucleotide background. Sets of mutation-type specific reference signatures have been developed and deposited in the Catalogue of Somatic Mutations in Cancer (COSMIC) database (Tate et al. 2019; Alexandrov et al. 2020; Sondka et al. 2023). These signatures were downloaded from the COSMIC website at https://cancer.sanger.ac.uk/signatures/downloads/. Code was written to combine these signatures linearly to best fit the observed AC25 counts for the 96 types, with coefficients constrained to be positive (by fitting the square roots of the coefficients). Those signatures for which the estimate of beta/SE exceeded 7 were retained in the model which was then refitted, to produce a parsimonious model to predict the observed AC25 counts from a small number of reference signatures.

### Modeling effects of background on variant frequencies

In order to allow for separate analyses of patterns of mutation related to transcription, the genome was divided into transcribed and nontranscribed regions. For the nontranscribed region variants were specified according to the plus strand whereas for transcribed regions variants were specified according to coding strand. The transcribed region was further subdivided between transcripts for protein coding genes and transcripts for noncoding RNA genes, since the coding genes are transcribed by RNA polymerase II while RNA genes are also synthesized by RNA polymerase I and III (Bunch 2018). There were 1,457,448 AC25 variants in the nontranscribed region and their frequencies correlated at *R* = 0.99 with the frequencies which had been observed in the DNM sample (Halldorsson et al. 2019). The frequencies also correlated at *R* = 0.99 with those which had been obtained for European subjects in the 1000 Genomes Project and which had been used for the earlier study of the effect of sequence context on substitution probabilities (Aggarwala and Voight 2016). These AC25 variants in the nontranscribed region were taken to result from mutations not due to transcription and were used for the analyses of effects of sequence context on mutation probabilities.

The general approach to analyzing sequence effects on mutation probabilities involved studying the frequency of observing a certain alternate allele conditional on the reference sequence background. For example, if the T nucleotide occurs 7.78e8 times in the reference genome and we observe 3.23e5 AC25 T > C variants then we say that the frequency of AC25 T > C variants occurring on the background of the T nucleotide is 3.23e5/7.78e8 = 0.000415. Likewise, if the trinucleotide sequence ATG occurs 4.55e7 times in the reference and there are 4.00e4 AC25 A[T > C]G variants then the frequency of AC25 A[T > C]G variants on the ATG background is 4.00e4/4.55e7 = 0.000880. Finally, if the pentanucleotide CATGC sequence occurs 3.89e6 times in the reference and there are 2,680 CA[T > C]GC AC25 variants then the frequency of CA[T > C]GC AC25 variants on the CATGC background is 2,680/3.89e6 = 0.000689.

Using this approach, it is possible to implement a logistic regression framework whereby the outcome to be predicted is a variant with a specific alternate base, referred to as the target, and this outcome is modeled as being influenced by the background reference sequence. Where background sequences are mutually exclusive, for example when considering all trinucleotides consisting of a central base and the flanking base on each side, one can simply total up counts across matching pentanucleotides to obtain the frequency for each background to produce a AC25 variant yielding a particular target base. This process was carried out for single nucleotides, dinucleotides including 1 upstream base, trinucleotides including 2 upstream or 2 flanking bases and tetranucleotides including 2 upstream and 1 downstream base.

In order to explore the effect on mutational probabilities of including the 2 flanking nucleotides relative to the effect of the core nucleotide, AC25 variant frequencies were obtained for background core nucleotides and then each flanking trinucleotide sequence was included in turn in a logistic regression model to estimate its additional effect on the odds of producing the target base. Similar analyses were done to measure the effects of models with the full pentanucleotide sequences relative to the flanking trinucleotide sequences.

### Analyzing effects related to transcription

The frequencies of AC25 variants in coding transcripts and noncoding transcripts both correlated with the frequencies for variants in nontranscribed regions with *R* > 0.995. However there were some variants for which frequencies were slightly but statistically significantly different. To investigate these differences, a logistic regression analysis was implemented to model whether the frequency of a variant differed between 1 region and another. Three comparisons were performed: between the nontranscribed region and protein coding gene region, between the nontranscribed region and RNA gene region and between the protein coding gene region and the RNA gene region. Analogously to the modeling of mutation probabilities, for each comparison modeling was performed using effects of single nucleotides and then incorporating varying numbers of flanking nucleotides up to the full pentanucleotide sequence.

A similar approach was used to model effects on strand asymmetries, with the outcome of interest being whether the variant occurred on the plus or minus strand for the nontranscribed region or whether it occurred on the coding or template strand for the transcribed regions.

In order to assess which genes were most affected by transcription related mutational processes, variants were identified which were more frequent in transcripts of protein coding genes than the nontranscribed region and which showed greater strand asymmetry in that they had higher frequency on the coding strand than the template strand. For these gene-based analyses, all variants were used rather than only those with an AC of 25. The 500 genes with the highest and lowest frequencies of these variants were entered into the PAN-GO gene ontology enrichment analysis to determine which biological processes they were involved with (https://functionome.geneontology.org/) (Mi et al. 2013).

### Data analysis

Code to count variants and background sequences as well as to carry out modeling was written in C++ and is available at: https://github.com/davenomiddlenamecurtis/countDNAVariants. This repository also contains raw counts and calculated frequencies of the variants analyzed. Additional data management and figure preparation was performed using R v4.3.2 (R Core Team 2014), including the *scico* package to utilize Fabio Crameri's scientific color maps (DOI 10.5281/zenodo.1243862).

## Results

### Mutational signature analysis

For the mutation signature analysis, the counts of standard 96 mutation types, consisting of all trinucleotide AC25 variants in which the core base was either C or T, were considered with the counts from the plus and minus strands being combined. As described in the Methods section, 86 reference signatures obtained from the COSMIC database were entered into a multiple linear regression analysis to obtain coefficients for a linear combination of signatures which would provide the best fit to the observed counts of AC25 variants (Tate et al. 2019). Coefficients were constrained to be positive and the intercept was constrained to zero. When this was done there were 7 signatures for which the ratio of the coefficient to its standard error exceeded 7 and these signatures were retained and the modeling repeated. These 7 signatures along with their best-fitting relative contributions are shown in Table 1. It should be noted that the proposed etiologies for these signatures as described in the COSMIC database do not necessarily reflect the mutational mechanisms which have produced them (Sondka et al. 2023). In particular, although the SBS87 signature can result from thiopurine chemotherapy treatment, in the present context we would interpret this as saying that SBS87 might result from similar biological mechanisms as those produced by thiopurine rather than resulting from actual exposure to thiopurine. The observed counts of mutation types and the predicted counts which would be obtained from combining these signatures according to the best-fitting weights have correlation coefficient *R* = 0.993. These observed and predicted counts are shown in Figs. 1 and 2.

**Fig. 1.**
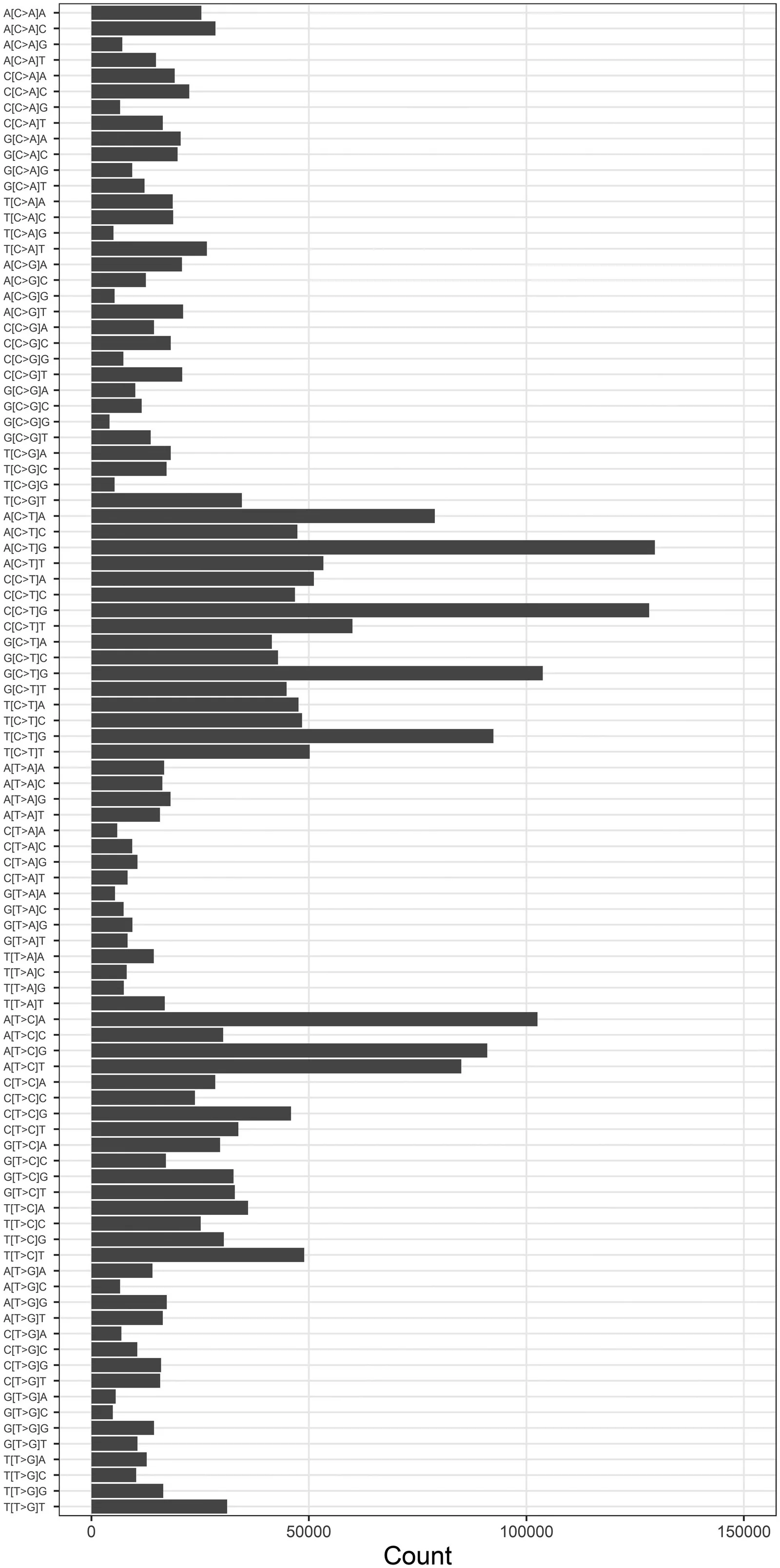
Observed counts of AC25 SBS variants on trinucleotide backgrounds.

**Fig. 2.**
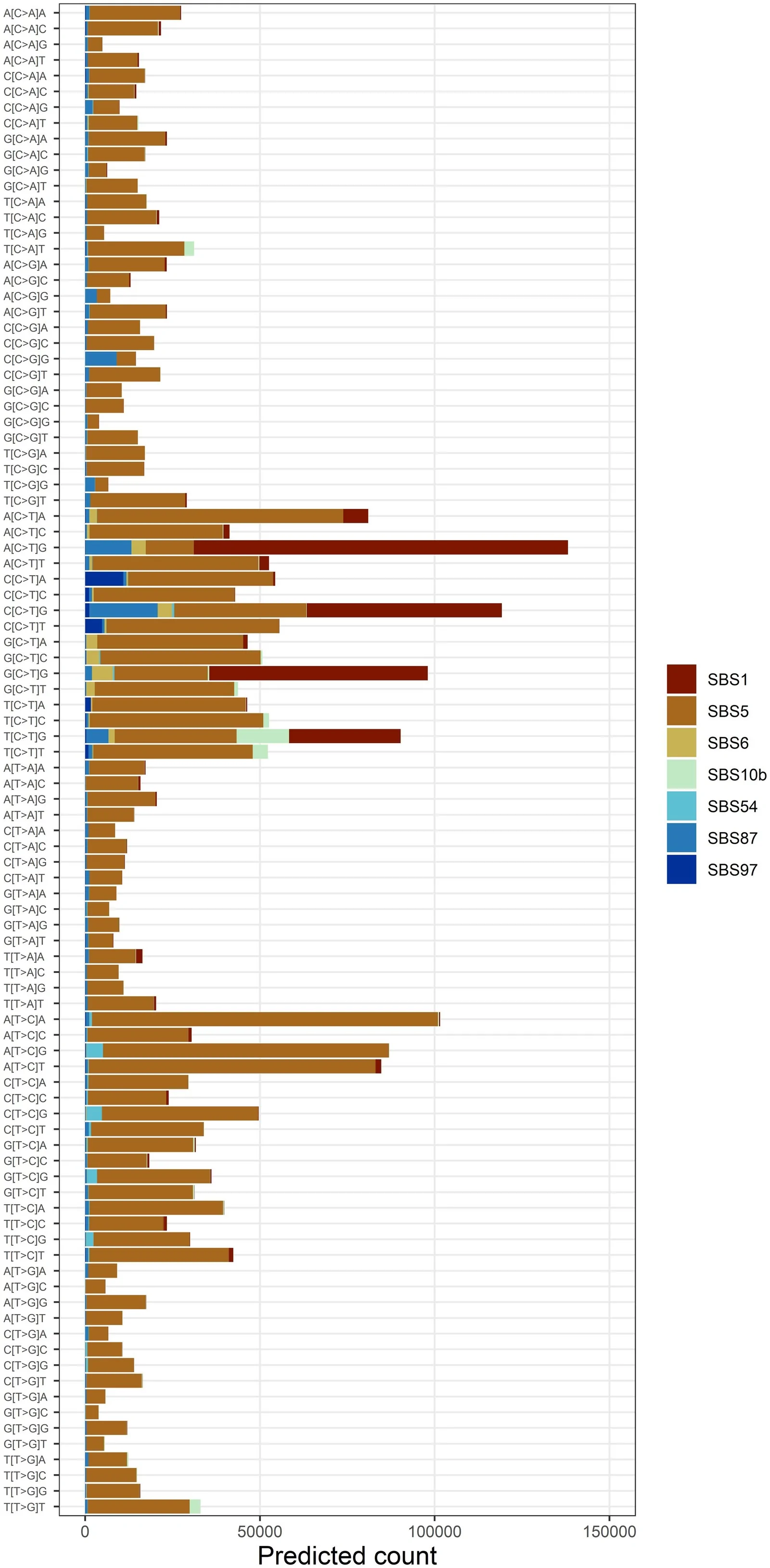
Predicted AC25 SBS counts using best-fitting combination of 7 reference mutational signatures obtained from studies of somatic mutations observed in cancers.

**Table 1.** Mutational signatures producing the best fit to observed counts of trinucleotide AC25 variants.

Signature	Relative contribution	Proposed etiology as described in COSMIC database
SBS1	0.178	Spontaneous or enzymatic deamination of 5-methylcytosine (clock-like signature)
SBS5	0.486	Unknown (clock-like signature)
SBS6	0.061	Defective DNA mismatch repair
SBS10b	0.061	Polymerase epsilon exonuclease domain mutations
SBS54	0.050	Possible sequencing artifact
SBS87	0.107	Thiopurine chemotherapy treatment
SBS97	0.057	Unknown

The mutational signatures are obtained from somatic mutations observed in cancer cells and their proposed etiologies are as stated for the COSMIC database at https://cancer.sanger.ac.uk/signatures/sbs/.

### Modeling effects of background on variant frequencies

Table 2 shows the frequencies of AC25 variants in the nontranscribed region observed on the background of a C or T nucleotide. It can be seen that the most frequent AC25 variant is C > T, with frequency 0.000919, and the rarest is T > A, with frequency 0.000113. AC25 variant frequencies for backgrounds including different numbers of flanking nucleotides were also calculated. For each partial background sequence, these frequencies could be used to predict the expected observed frequencies for each AC25 variant in the context of the full pentanucleotide background. If just the core nucleotide was used then the predicted frequencies correlated with the observed frequencies with *R* = 0.508. If a single nucleotide upstream was included the correlation increased to *R* = 0.746 and including the second upstream nucleotide yielded *R* = 0.747, while including the 2 flanking nucleotides yielded *R* = 0.992. Finally, if 2 upstream and 1 downstream nucleotide were included the correlation was R = 0.995. These results suggest that a major driver for the frequency of each variant is the probability of the central base substitution itself but that this probability is strongly influenced by the 2 neighboring nucleotides.

**Table 2.** The table shows frequencies for the 6 different single nucleotide variants having C or T as the background (reference) base relative to the counts of the reference base for AC25 variants in the nontranscribed region of the genome.

Substitution	Frequencies of AC25 variants
C > A	0.000256
C > G	0.000204
C > T	0.000919
T > A	0.000113
T > C	0.000418
T > G	0.000129

Logistic regression modeling was used to better understand which features of the 2 flanking nucleotides had most effect on the frequency. Figure 3 shows the frequencies of all AC25 variants occurring on trinucleotide sequences with either C or T as the central base. Figure 4 shows the odds ratio (OR) for each trinucleotide sequence to produce the target base relative to the odds for just the central base. The graph does not show the results for variant types having the pattern X[C > T]G because these all had very large ORs compared to the others, with ORs of 8.87, 9.36, 10.17, and 11.16 for T[C > T]G, G[C > T]G, C[C > T]G, and A[C > T]G respectively. Among the other variant types, the 2 for which the flanking nucleotides have the strongest positive effect are A[T > C]A and A[T > C]G with ORs of 2.26 and 2.24, respectively. Conversely T[C > T]T and T[C > T]A have ORs of 0.421 and 0.448, indicating that these flanking nucleotides act to reduce the probability of the variant occurring. Results for all trinucleotide backgrounds having C or T as the central base are presented in Supplementary Table 1.

**Fig. 3.**
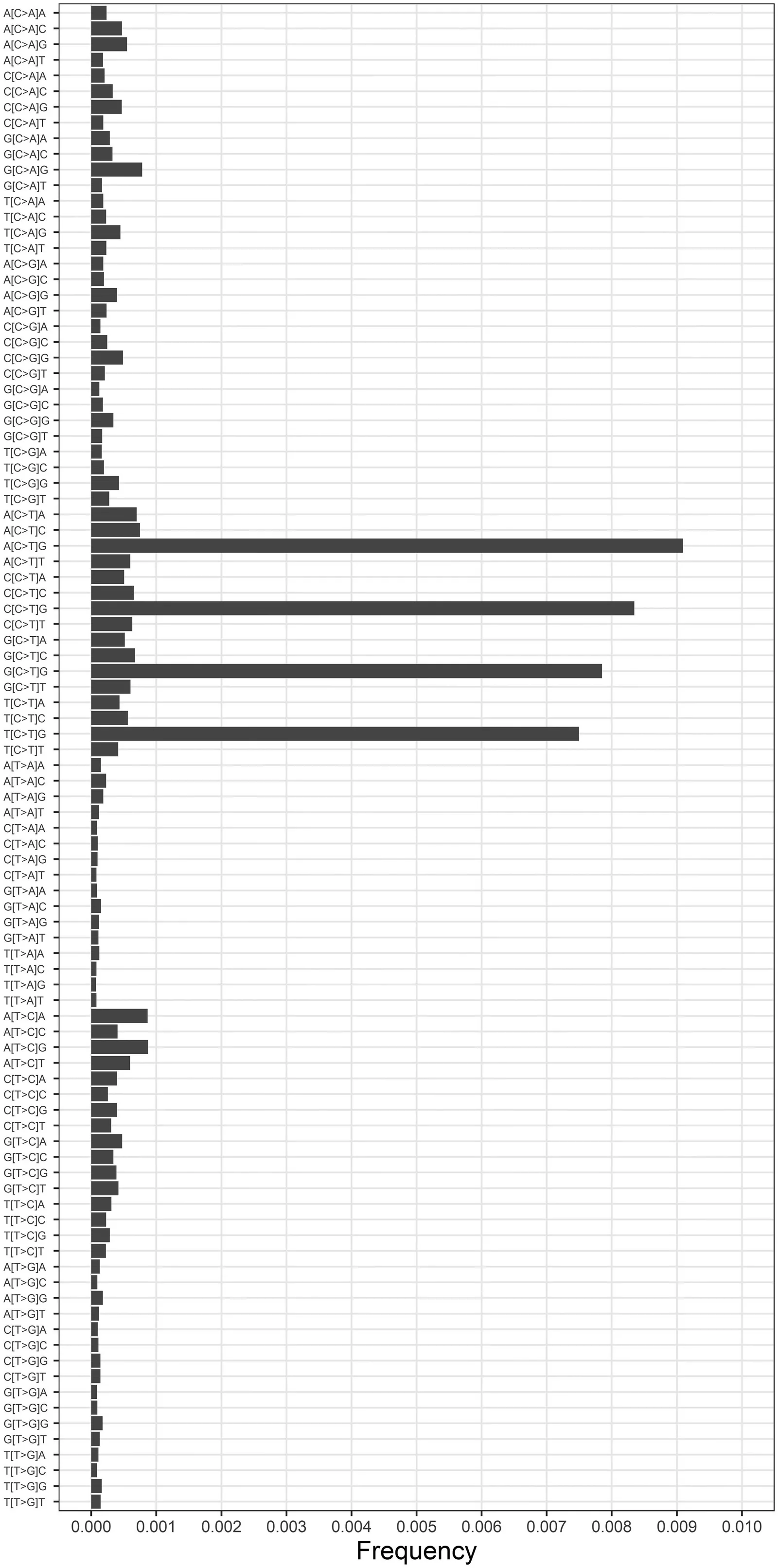
Frequencies of all AC25 variants occurring in the context of different trinucleotide backgrounds. The frequency is defined as the count of the number of times each AC25 variant is observed divided by the count of the number of times the background is seen in the reference genome.

**Fig. 4.**
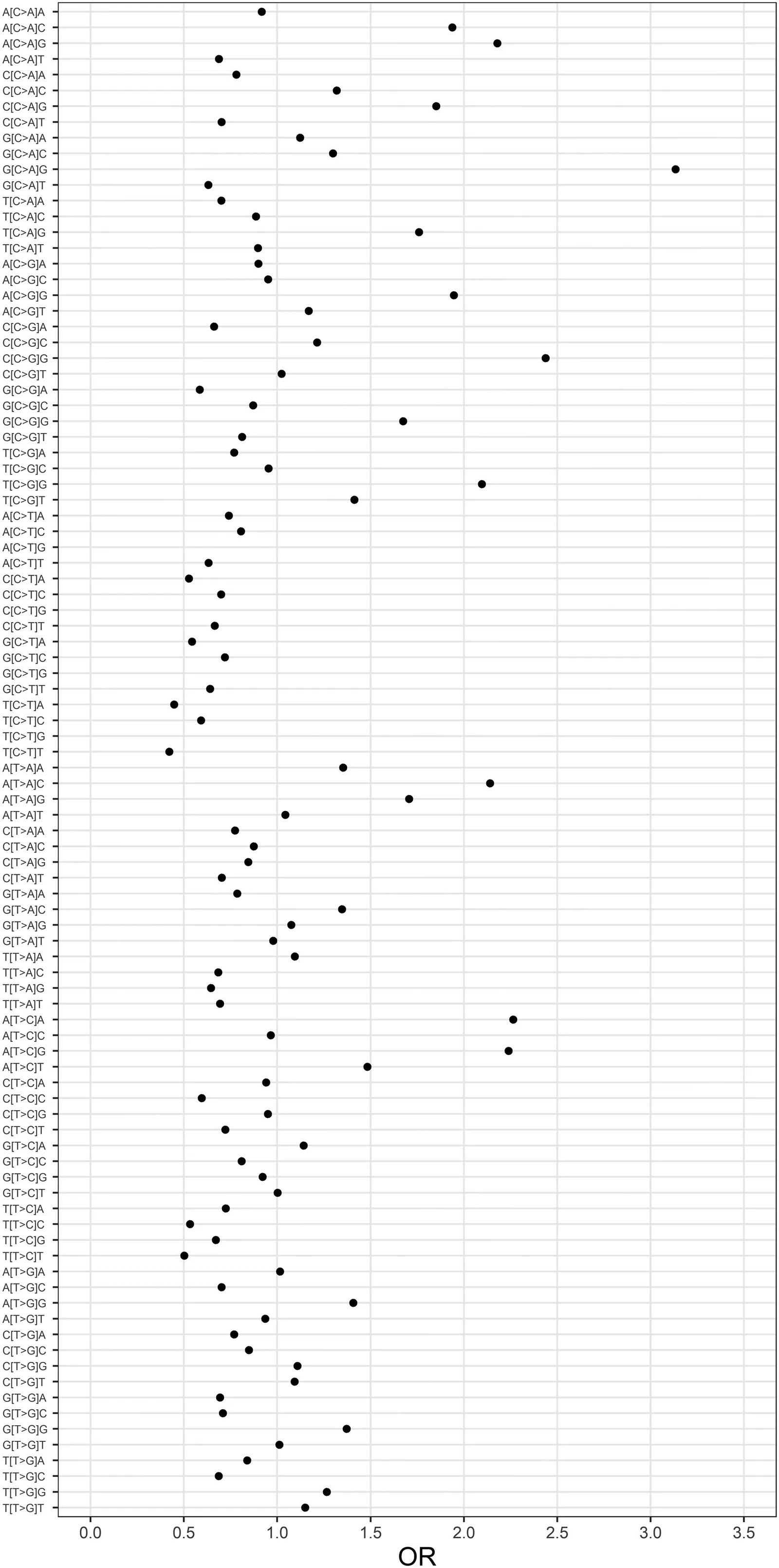
Odds ratio (OR) for each AC25 variant associated with its trinucleotide background, defined as the frequency with which the variant occurs on this background compared with the overall frequency of AC25 single base substitutions of this type (e.g. frequency of A[T > A]C vs frequency of T > A). The results for X[C > T]G variants are not shown because the ORs are too high.

To evaluate the effects of the full pentanucleotide background compared to the trinucleotide background, the modeling procedure was repeated to obtain the OR for each pentanucleotide sequence to produce the target base relative to the odds of the central trinucleotide sequence. Table 3 shows the variant types with the 10 lowest and highest ORs. These results indicate that the more distant nucleotides can have major effects. For example, if there is a C upstream and downstream then the frequency of G[C > A]A and G[C > A]G variants is reduced by a factor of more than 2. Similar results are seen for C[C > G]A variants if there is a T upstream and a C or T downstream. Conversely, TT[T > A]AA and CA[T > C]TG variants have a 4-fold higher frequency than the mean for T[T > A]A and A[T > C]T variants. For A[T > C]T variants, having a G downstream and a C, G or T upstream more than doubles the frequency. Results for all pentanucleotide backgrounds having C or T as the central base are presented in Supplementary Table 2.

**Table 3.** Table showing the effect of the full pentanucleotide sequence vs the central trinucleotide sequence on the frequencies of AC25 variants in the nontranscribed region.

Variant	OR	(0.95 CI)
CG[C > A]AC	0.281	(0.171 to 0.464)
CG[C > A]GC	0.371	(0.266 to 0.516)
TC[C > G]AC	0.382	(0.305 to 0.478)
CG[C > A]GA	0.395	(0.243 to 0.643)
TC[C > A]CA	0.408	(0.368 to 0.453)
TA[T > A]CG	0.408	(0.234 to 0.710)
CT[T > A]AG	0.415	(0.321 to 0.536)
GC[C > A]CG	0.417	(0.311 to 0.561)
TC[C > G]AT	0.419	(0.350 to 0.502)
GA[T > A]CG	0.434	(0.277 to 0.679)
TA[T > C]TG	2.458	(2.358 to 2.563)
CC[C > G]CC	2.502	(2.286 to 2.739)
GA[T > G]AG	2.586	(2.303 to 2.903)
CG[C > G]GA	2.621	(1.945 to 3.532)
AA[T > C]TG	2.707	(2.603 to 2.815)
GA[T > C]TG	2.804	(2.672 to 2.943)
CC[T > G]TC	2.821	(2.591 to 3.071)
CC[C > A]CC	3.326	(3.101 to 3.567)
CA[T > C]TG	3.947	(3.805 to 4.094)
TT[T > A]AA	4.345	(4.071 to 4.637)

Results are shown for the 10 variants with T or C as the central base for which the frequency is most strongly reduced or increased.

### Analyzing effects related to transcription

The frequencies of AC25 variants in protein coding transcripts and RNA gene transcripts both correlated with the frequencies for variants in nontranscribed regions with *R* > 0.995. Nevertheless, the absolute and relative frequencies did differ to some extent. Table 4 displays the differences in frequencies of single nucleotide variants between the different regions. This shows for example that many variants are somewhat less frequent in transcripts of protein coding genes than in the nontranscribed region but that A > G variants are somewhat commoner on the coding strand of protein coding gene transcripts than they are in the nontranscribed region, with OR 1.204 (1.196 to 1.213). However the same variant occurs less commonly on the template strand since the OR for T > C variants is 0.775 (0.768 to 0.781). The same effects can be seen to be present, though less strongly, for transcripts of RNA genes compared to the nontranscribed region, with corresponding ORs of 1.082 (1.071 to 1.093) and 0.907 (0.897 to 0.917). The ORs for these frequencies between RNA gene and protein coding transcripts have narrow confidence intervals, 0.898 (0.889 to 0.908) and 1.171 (1.158 to 1.185), suggesting that these differences are nonrandom.

**Table 4.** The table shows the ORs of frequencies for AC25 variants between 3 different regions of the genome, consisting of the nontranscribed region, the transcripts of protein coding genes and the transcripts of RNA genes.

Variant	OR (0.95 CI)					
	Protein coding vs nontranscribed		RNA gene vs nontranscribed		RNA gene vs protein coding	
T > C	0.775	(0.768 to 0.781)	0.907	(0.897 to 0.917)	1.171	(1.158 to 1.185)
G > C	0.863	(0.851 to 0.874)	0.952	(0.935 to 0.969)	1.103	(1.082 to 1.125)
A > C	0.921	(0.908 to 0.934)	0.975	(0.957 to 0.994)	1.059	(1.037 to 1.081)
C > T	1.051	(1.044 to 1.057)	0.998	(0.989 to 1.006)	0.950	(0.941 to 0.958)
G > T	0.856	(0.846 to 0.866)	0.967	(0.952 to 0.983)	1.130	(1.111 to 1.150)
A > T	0.927	(0.914 to 0.941)	0.957	(0.938 to 0.977)	1.032	(1.010 to 1.055)
C > G	1.084	(1.071 to 1.098)	1.038	(1.020 to 1.057)	0.958	(0.940 to 0.976)
T > G	0.935	(0.922 to 0.948)	0.975	(0.957 to 0.993)	1.043	(1.023 to 1.064)
A > G	1.204	(1.196 to 1.213)	1.082	(1.071 to 1.093)	0.898	(0.889 to 0.908)
C > A	0.720	(0.711 to 0.730)	0.903	(0.888 to 0.918)	1.254	(1.231 to 1.277)
T > A	0.778	(0.766 to 0.790)	0.908	(0.889 to 0.926)	1.166	(1.140 to 1.193)
G > A	0.981	(0.975 to 0.987)	0.970	(0.962 to 0.978)	0.989	(0.980 to 0.998)

For the nontranscribed region variants are defined by the plus strand and for the transcribed region variants are defined by the coding strand.

Similarly to the analyses of effects of sequence context on mutation probability, logistic regression modeling was used to determine how strongly subsets numbers of nucleotides predicted the ORs of frequencies of pentanucleotide variants between different regions. The correlation coefficients for these predictions are presented in Table 5. In general, there are 2 notable findings. One is that, when predicting ORs relative to nontranscribed regions, the correlation coefficients are lower for the RNA genes than for the protein coding genes. The other is that the correlation coefficients for the protein coding genes are considerably lower than were observed for predicting sequence effects on raw mutation frequencies. For example, the frequencies for the central trinucleotide predicted the raw pentanucleotide frequencies in the nontranscribed region with *R* = 0.992 but the trinucleotide ORs for frequency differences between the nontranscribed and protein coding region predict the observed pentanucleotide ORs with *R* = 0.602. An interpretation of this might be that the relative probability for a mutation to occur in the nontranscribed region vs a protein coding transcript is not strongly determined by the immediately flanking nucleotides, as defined by the trinucleotide, but is more dependent on the full pentanucleotide context.

**Table 5.** The table shows the correlation coefficient between the ORs of observed frequencies of full pentanucleotide AC25 variants between regions and the ORs predicted from using a subset of the component nucleotides.

Nucleotides included	Correlation coefficient between predicted and actual pentanucleotide variant frequency ORs		
	Protein coding vs nontranscribed	RNA gene vs nontranscribed	RNA gene vs protein coding
Central nucleotide only	0.536	0.200	0.312
Central and 1 upstream	0.581	0.228	0.355
Central and 1 downstream	0.580	0.221	0.348
Central and 2 upstream	0.643	0.287	0.414
Central and 2 downstream	0.630	0.294	0.416
Central and 2 flanking	0.602	0.279	0.393
Central, 2 upstream, 1 downstream	0.744	0.491	0.564
Central, 1 upstream, 2 downstream	0.713	0.485	0.566

Table 6 shows the 20 variants which have the most significantly increased frequencies in protein coding and RNA gene transcripts compared to the nontranscribed region, as assessed by having the largest values for *z* = ln(OR)/SE which under the null hypothesis follows a Gaussian distribution with zero mean and unit standard deviation. Strikingly, it can be seen that the 8 variants with the strongest evidence for increased frequencies in transcripts of protein coding genes all have the motif CT[A > G]T* or CA[A > G]T* and these all have OR > 1.5. All of the top 20 variants have the A > G substitution. For the RNA gene transcripts effect sizes are smaller and less strongly statistically significant but it seems that similar effects operate in that the top 4 variants match one of these motifs and all but one of the top 20 has the A > G substitution. To explore whether there were systematic differences between the protein coding gene transcripts and the RNA gene transcripts, their variant frequencies were compared directly and the results are shown in Table 7. This shows that, for example, variants matching the CT[A > G]T* and CA[A > G]T* are significantly more frequent in protein coding gene transcripts than in RNA gene transcripts. Conversely there are variants which are more common in RNA gene transcripts than protein coding gene transcripts and these include some with motifs *A[T > C]AG and *A[T > C]TG. Notably, these motifs are complementary to the CT[A > G]T* and CA[A > G]T* motifs, meaning that another way to express these findings would be to say that CT[A > G]T* and CA[A > G]T* variants on the coding strand are more frequent in protein coding than RNA gene transcripts but on the template strand they are more frequent in RNA gene than protein coding gene transcripts.

**Table 6.** Table showing the variants with the strongest evidence for having a higher frequency in transcripts of protein coding genes and RNA genes relative to their frequency in the nontranscribed region.

Comparison of transcripts of protein coding genes with nontranscribed region.			
Variant	OR	(0.95 CI)	*z* = ln(OR)/SE
CT[A > G]TG	1.793	(1.692 to 1.900)	20.13
CT[A > G]TT	1.696	(1.604 to 1.794)	18.91
CA[A > G]TT	1.655	(1.565 to 1.749)	18.18
CA[A > G]TG	1.586	(1.505 to 1.671)	17.64
CA[A > G]TC	1.834	(1.711 to 1.965)	17.52
CA[A > G]TA	1.623	(1.527 to 1.725)	15.93
CT[A > G]TA	1.604	(1.511 to 1.702)	15.88
CT[A > G]TC	1.632	(1.512 to 1.761)	12.86
CC[A > G]TG	1.515	(1.417 to 1.621)	12.38
CA[A > G]AT	1.609	(1.473 to 1.759)	10.73
CA[A > G]CT	1.592	(1.458 to 1.739)	10.55
AT[A > G]TA	1.301	(1.236 to 1.368)	10.36
CA[A > G]CC	1.685	(1.511 to 1.878)	9.60
GT[A > G]TG	1.519	(1.384 to 1.666)	9.02
AC[A > G]TG	1.329	(1.248 to 1.417)	8.98
CT[A > G]CT	1.520	(1.382 to 1.671)	8.82
CA[A > G]AG	1.557	(1.407 to 1.722)	8.78
CC[A > G]TA	1.384	(1.284 to 1.491)	8.71
AC[A > G]TA	1.305	(1.227 to 1.388)	8.63
CA[A > G]AA	1.406	(1.298 to 1.523)	8.51

Comparison of transcripts of RNA genes with nontranscribed region.			
Variant	OR	(0.95 CI)	*z* = ln(OR)/SE
CT[A > G]TA	1.345	(1.241 to 1.459)	7.32
CA[A > G]TT	1.232	(1.141 to 1.330)	5.45
CT[A > G]TG	1.234	(1.134 to 1.341)	5.01
CA[A > G]TC	1.266	(1.148 to 1.396)	4.81
CC[A > G]TG	1.255	(1.140 to 1.381)	4.74
CA[A > G]TG	1.169	(1.087 to 1.257)	4.31
GT[A > G]TA	1.233	(1.117 to 1.361)	4.24
CA[A > G]CT	1.281	(1.134 to 1.446)	4.07
CT[A > G]TT	1.171	(1.080 to 1.271)	3.90
AC[A > G]TA	1.171	(1.078 to 1.272)	3.81
CA[A > G]CA	1.244	(1.106 to 1.399)	3.71
TC[A > G]TG	1.217	(1.091 to 1.358)	3.60
TT[A > T]CA	1.766	(1.276 to 2.444)	3.50
TT[G > A]TG	1.228	(1.083 to 1.391)	3.27
CT[C > G]AA	1.306	(1.103 to 1.547)	3.16
TT[A > C]AG	1.513	(1.162 to 1.971)	3.14
TG[C > G]AA	1.46	(1.137 to 1.875)	3.03
CT[A > G]TC	1.178	(1.057 to 1.313)	3.03
CT[C > G]TC	1.214	(1.065 to 1.382)	2.97
TT[C > T]GT	1.154	(1.047 to 1.272)	2.94

**Table 7.** Table showing the variants with the strongest evidence for having a higher frequency in transcripts of protein coding genes relative to their frequency in transcripts of RNA genes or vice versa.

Variants with higher frequency in transcripts of protein coding genes than transcripts of RNA genes.			
Variant	OR	(0.95 CI)	*z* = ln(OR)/SE
CT[A > G]TT	1.448	(1.334 to 1.572)	9.01
CT[A > G]TG	1.454	(1.336 to 1.582)	8.87
CA[A > G]TA	1.435	(1.313 to 1.569)	8.10
CA[A > G]TG	1.357	(1.258 to 1.463)	8.08
CA[A > G]TT	1.343	(1.241 to 1.453)	7.46
CA[A > G]TC	1.449	(1.310 to 1.602)	7.38
CT[A > G]TC	1.386	(1.240 to 1.548)	5.88
AT[A > G]TA	1.206	(1.120 to 1.299)	5.03
CA[A > G]AT	1.366	(1.201 to 1.555)	4.83
CA[A > G]GG	1.592	(1.311 to 1.932)	4.80
CA[A > G]AG	1.425	(1.223 to 1.659)	4.64
CA[A > G]AA	1.323	(1.172 to 1.493)	4.63
CA[A > G]CC	1.463	(1.240 to 1.726)	4.60
GT[A > G]TG	1.346	(1.173 to 1.546)	4.31
CT[A > G]TA	1.192	(1.097 to 1.295)	4.22
TT[A > G]TA	1.230	(1.111 to 1.361)	4.07
AT[A > G]TG	1.254	(1.122 to 1.402)	4.06
CC[A > G]TA	1.249	(1.119 to 1.393)	4.05
AC[A > G]TG	1.205	(1.098 to 1.323)	4.00
CA[A > G]GT	1.413	(1.185 to 1.684)	3.94

Variants with higher frequency in transcripts of RNA genes than transcripts of protein coding genes.			
Variant	OR	(0.95 CI)	*z* = ln(OR)/SE
CA[T > C]AG	0.673	(0.601 to 0.754)	−6.96
CA[T > C]TG	0.750	(0.688 to 0.818)	−6.60
AA[C > A]AA	0.634	(0.545 to 0.738)	−6.03
AA[T > C]AG	0.735	(0.662 to 0.816)	−5.91
CA[T > C]AT	0.686	(0.601 to 0.784)	−5.65
GA[T > C]AG	0.683	(0.595 to 0.784)	−5.54
GA[G > C]AG	0.658	(0.562 to 0.771)	−5.31
CA[T > C]GG	0.737	(0.655 to 0.828)	−5.21
TG[T > C]TG	0.693	(0.601 to 0.798)	−5.18
AA[T > C]GG	0.719	(0.633 to 0.818)	−5.13
AA[T > C]TG	0.780	(0.706 to 0.861)	−5.02
CA[T > A]CT	0.559	(0.442 to 0.708)	−4.94
TG[C > T]CT	0.761	(0.676 to 0.856)	−4.65
GA[T > C]AA	0.662	(0.554 to 0.791)	−4.64
AA[C > A]TT	0.571	(0.448 to 0.727)	−4.63
AT[T > C]TG	0.715	(0.618 to 0.828)	−4.58
TG[C > T]TT	0.750	(0.660 to 0.854)	−4.45
TA[C > A]TC	0.380	(0.245 to 0.588)	−4.43
AT[C > A]CA	0.619	(0.498 to 0.770)	−4.40
CA[T > A]CC	0.558	(0.426 to 0.730)	−4.35

Strand asymmetry of variants was assessed directly in the 3 regions by comparing the frequency of each variant against the complementary sequence or, putting it another way, the frequency of the variant observed relative to the other strand. Results for the variants showing the strongest evidence for asymmetry are shown in Table 8. In the nontranscribed region effects are small, nonsystematic and mostly not statistically significant. By contrast, in the transcripts of protein coding genes the CT[A > G]T* and CA[A > G]T* variants all occur more commonly on the coding strand than template strand with ORs exceeding 2. Other variants also exhibit marked asymmetry and all those in the top 20 have the A > G substitution. For the transcripts of RNA genes the effects seem similar though less marked.

**Table 8.** Tables showing the variants with the strongest evidence for strand asymmetry.

a. Variants with higher frequency on the plus vs minus strand of the nontranscribed region.			
Variant	OR	(0.95 CI)	*z* = ln(OR)/SE
CC[G > A]TT	1.173	(1.096 to 1.257)	4.67
TG[T > A]AA	1.563	(1.246 to 1.962)	3.93
TT[T > G]GT	1.248	(1.110 to 1.403)	3.78
CG[T > C]TA	2.745	(1.606 to 4.693)	3.77
TC[T > C]TC	1.211	(1.091 to 1.345)	3.68
CC[C > G]AG	1.253	(1.089 to 1.443)	3.20
TA[G > A]CA	1.145	(1.051 to 1.248)	3.16
TC[G > T]GG	1.515	(1.161 to 1.976)	3.12
GT[T > G]CT	1.552	(1.171 to 2.056)	3.12
AT[A > C]AA	1.260	(1.084 to 1.465)	3.06
GA[C > G]AT	1.450	(1.129 to 1.862)	2.97
GA[T > C]TT	1.152	(1.046 to 1.269)	2.93
TG[C > A]CC	1.211	(1.061 to 1.383)	2.90
CT[A > G]GC	1.253	(1.069 to 1.470)	2.84
GT[A > C]TG	1.529	(1.132 to 2.066)	2.82
AG[C > A]TC	1.313	(1.081 to 1.596)	2.80
AG[T > A]CT	1.244	(1.062 to 1.456)	2.77
CA[A > C]AA	1.193	(1.050 to 1.355)	2.76
AC[G > C]AT	1.526	(1.117 to 2.086)	2.71
TG[T > G]GT	1.193	(1.046 to 1.359)	2.69

b. Variants with higher frequency on the coding vs template strand in transcripts of protein coding genes.			
Variant	OR	(0.95 CI)	*z* = ln(OR)/SE
CT[A > G]TG	3.150	(2.901 to 3.419)	27.94
CA[A > G]TG	2.273	(2.129 to 2.426)	25.13
CA[A > G]TT	2.428	(2.259 to 2.611)	24.49
CT[A > G]TT	2.513	(2.329 to 2.711)	24.23
CA[A > G]TC	2.439	(2.241 to 2.654)	21.09
CT[A > G]TA	2.039	(1.895 to 2.194)	19.42
CT[A > G]TC	2.653	(2.395 to 2.938)	19.11
CA[A > G]TA	2.011	(1.868 to 2.163)	19.06
CC[A > G]TG	2.049	(1.882 to 2.231)	16.85
AT[A > G]TA	1.656	(1.554 to 1.764)	15.92
CA[A > G]CA	2.251	(2.022 to 2.507)	15.11
CA[A > G]AT	2.269	(2.029 to 2.536)	14.70
AT[A > G]TG	2.085	(1.882 to 2.311)	14.30
CA[A > G]CT	2.115	(1.903 to 2.352)	14.14
CA[A > G]CC	2.562	(2.233 to 2.939)	13.70
CT[A > G]CT	2.179	(1.939 to 2.449)	13.34
GT[A > G]TG	2.230	(1.964 to 2.532)	12.64
CC[A > G]TT	1.853	(1.681 to 2.044)	12.63
AC[A > G]TG	1.572	(1.458 to 1.695)	12.05
GC[A > G]TG	1.811	(1.640 to 2.001)	11.96

c. Variants with higher frequency on the coding vs template strand in transcripts of RNA genes.			
Variant	OR	(0.95 CI)	*z* = ln(OR)/SE
CT[A > G]TA	1.466	(1.314 to 1.636)	6.98
CA[A > G]TT	1.410	(1.271 to 1.565)	6.62
CT[A > G]TG	1.458	(1.299 to 1.636)	6.53
GT[A > G]TA	1.512	(1.315 to 1.737)	5.95
CA[A > G]TG	1.257	(1.143 to 1.382)	4.81
CT[C > G]AA	1.730	(1.361 to 2.198)	4.58
CT[A > G]TT	1.275	(1.144 to 1.422)	4.47
CA[A > G]TA	1.275	(1.140 to 1.426)	4.34
CA[A > G]TC	1.320	(1.161 to 1.501)	4.33
CA[A > G]CA	1.370	(1.174 to 1.599)	4.07
AT[A > G]TA	1.206	(1.099 to 1.324)	4.02
TC[A > G]TG	1.320	(1.141 to 1.529)	3.80
CA[A > G]CT	1.346	(1.148 to 1.580)	3.72
AC[A > G]TA	1.220	(1.096 to 1.359)	3.72
CT[A > G]TC	1.307	(1.131 to 1.510)	3.71
CA[G > T]TT	1.759	(1.293 to 2.394)	3.67
AG[A > G]TG	1.387	(1.157 to 1.663)	3.61
CC[A > G]TG	1.250	(1.102 to 1.417)	3.54
CT[A > C]TA	1.866	(1.307 to 2.666)	3.50
CT[A > G]CA	1.365	(1.142 to 1.630)	3.49

The detailed results for all comparisons of variant frequencies between regions and between strands are provided in Supplementary Tables 3 and 4.

In order to investigate which genes contributed to these effects, the frequencies of all CT[A > G]T* variants (not just those with AC = 25) were calculated for each protein coding gene with length ≥ 15 kb. The 500 genes with the highest and lowest frequencies for these variants were entered into a PAN-GO enrichment analysis for biological pathways and the results are displayed in Table 9. Table 9 shows that the genes with the highest frequencies of these variants tend to be involved in gene expression and RNA metabolism whereas the second part of Table 9 shows that genes with the lowest variant frequencies are less likely to be involved in these processes. Instead, the second part of Table 9 shows that the genes with the lowest variant frequencies demonstrate enrichment for more specialized processes such as synaptic transmission and muscle contraction.

**Table 9.** Results of PAN-GO enrichment analyses for genes with the highest and lowest frequencies of CT[A>G]T* variants showing the top level biological processes with statistically significant enrichment at false discovery rate (FDR) < 0.05.

Results for 500 genes with highest frequencies of CT[A > G]T* variants.			
PAN-GO biological process	Fold enrichment	Raw *P*-value	FDR
regulation of mRNA metabolic process (GO:1903311)	3.75	8.93E−05	2.56E−02
DNA-templated transcription (GO:0006351)	3.49	1.81E−04	4.99E−02
positive regulation of RNA metabolic process (GO:0051254)	2.57	5.61E−05	1.93E−02
macromolecule catabolic process (GO:0009057)	2.17	5.70E−05	1.87E−02
regulation of gene expression (GO:0010468)	1.54	1.46E−05	7.75E−03
multicellular organismal process (GO:0032501)	0.37	1.12E−06	1.55E−03

Results for 500 genes with lowest frequencies of CT[A > G]T* variants.			
PAN-GO biological process	Fold enrichment	Raw *P*-value	FDR
detection of bacterium (GO:0016045)	43.24	1.23E−05	6.05E−03
G protein-coupled receptor signaling pathway, coupled to cyclic nucleotide second messenger (GO:0007187)	9.83	1.23E−04	2.74E−02
muscle contraction (GO:0006936)	5.33	4.59E−05	1.37E−02
regulation of membrane potential (GO:0042391)	3.90	2.56E−04	4.89E−02
organic anion transport (GO:0015711)	3.82	5.06E−06	4.36E−03
chemical synaptic transmission (GO:0007268)	3.80	5.43E−06	4.16E−03
monoatomic ion transmembrane transport (GO:0034220)	2.61	3.04E−05	1.05E−02
inorganic cation transmembrane transport (GO:0098662)	2.59	2.35E−04	4.91E−02
organelle organization (GO:0006996)	0.41	5.16E−05	1.42E−02
gene expression (GO:0010467)	0.37	2.54E−04	4.99E−02
regulation of transcription by RNA polymerase II (GO:0006357)	0.36	5.02E−06	4.94E−03
RNA biosynthetic process (GO:0032774)	0.14	1.05E−04	2.50E−02

A “Fold enrichment” value <1 indicates a depletion of genes involved in the relevant pathway.

## Discussion

Unless they occur as recurrent mutations, DNMs occurring in research participants within a sample will appear as singleton variants and it might seem tempting to use singleton variants to gain information about mutation rates. However in this large cohort each participant carries around 900 singleton variants and is expected to have only 60 or so DNMs, so the vast majority of singleton variants consist of rare variants segregating in the population rather than DNMs. Moreover, as discussed previously, singleton counts can be reduced for variant types with high mutation rates so that overall the counts of singleton variants do not accurately reflect mutation probabilities (Wakeley et al. 2023). Instead, we show that if one considers variants each of which has an AC of 25, rather than 1, then one finds that the frequencies of the AC25 variant types are very highly correlated with the frequencies of DNMs directly observed in a previously reported, smaller sample (Halldorsson et al. 2019). This finding applies to the present sample of 500,000 genomes and other AC choices might be more appropriate for other samples.

The large sample size allows for the study of sequence-based effects on relative mutation rates in a normal population. Studying the rates of different mutation types in spermatozoa provides information mainly concerning errors occurring during spermatogenesis (Shoag et al. 2025). Studying cancer cells provides information about somatic mutations which have arisen either during mitosis or at some other point in the cell cycle but which have occurred in the context of disease, when the normal mechanisms for maintaining and accurately copying DNA have been disrupted (Alexandrov et al. 2020). By contrast, AC25 variants observed in a large population sample are expected to reflect mutational events which have occurred in essentially healthy cells. They may have arisen during meiosis or as somatic mutations in primordial germ cells. Thus, studying the relative frequency of different types of AC25 variant may throw light on the molecular mechanisms used by healthy cells to prevent mutations from occurring and this may lead to better insights into susceptibility to neoplastic disease.

We show that the counts of these AC25 variants, which we take to reflect mutation rates, when considered in a trinucleotide context can be very accurately modeled using a small number of mutational signatures which had been obtained from analyzing somatic mutations in cancer tissue. The signature with the largest contribution, SBS5, has been described as clock-like, in that the number of these mutations increases with the age of the individual, and may be due to DNA damage to adenine (Alexandrov et al. 2020). It has been reported to dominate among DNMs as well as in somatic cells, cultured cells and tumors and appears to result from multiple types of DNA damage (Spisak et al. 2025 Sep 1). Likewise, the signature with the second largest contribution, SBS1, is also clock-like and may reflect deamination of methylcytosine (Silveira et al. 2024). Comparing these AC25 variant frequencies with those observed in cancer will hopefully facilitate a fuller understanding of pathways to carcinogenesis.

Using variants in the nontranscribed region, we replicate the previously reported finding that the frequency of C > T variants is much increased in the CpG context but we also demonstrate how other aspects of the context can have quite marked effects on variant frequency and hence, we assume, mutation rate (Zhao and Boerwinkle 2002). Although the trinucleotide context largely predicts mutation rate, we demonstrate that in some situations the fully pentanucleotide context has relevance and we see examples where the more distant bases have 2-fold or higher impacts. Investigations into why these specific bases have the effects that they do could lead to a better understanding of the molecular mechanisms involved in generating these mutations.

For some variants, the frequencies differ between nontranscribed and transcribed regions. Possibly this could arise from differences in the organization of the regions, including features such as repeat content, but these frequency differences might also be the result of mutational processes occurring during transcription. CT[A > G]T* and CA[A > G]T* variants are more frequent in the transcripts of protein coding genes than in the nontranscribed region and occur more frequently on the coding strand than the template strand. The genes with the highest frequencies of CT[A > G]T* variants show enrichment for gene expression and RNA metabolism processes which would be active in all cells, while the genes with the lowest frequencies of these variants show enrichment for processes active only in specialized cells, such as neurons and myocytes. These findings all tend to support the notion that these variants can arise during transcriptional activity and if they occur during embryogenesis or gametocyte differentiation they can then go on to enter the germline.

Some variants differ in frequency between transcripts of protein coding genes and RNA genes. This might reflect differences in genome organization or transcription levels but such differences might also result from differential effects of the transcription machinery involved in different types of gene.

A more detailed analysis of variant frequencies associated with different types of transcript will be the subject of further work. It will also be fruitful to investigate the distribution of frequencies of variants which have been implicated in human disease. Having a fuller understanding of the mechanisms which can lead to variant formation and how they are affected by context and location in the genome may assist in improving our ability to predict expected effects and pathogenicity.

## Supplementary Material

jkag207_Supplementary_Data

## Data Availability

The raw data are available on application to UK Biobank at https://ams.ukbiobank.ac.uk/ams/. Any legitimate researcher can apply for access to the UK Biobank dataset by filling in the form at this URL. The counts of variants and of pentanucleotide sequences in the reference genome are provided at https://github.com/davenomiddlenamecurtis/countDNAVariants. Supplemental material available at G3 online.
